# Numerical Study of a Thrombus Migration Risk in Aneurysm After Coil Embolization in Patient Cases: FSI Modelling

**DOI:** 10.1007/s13239-023-00672-4

**Published:** 2023-07-19

**Authors:** C. Paz, E. Suárez, A. Cabarcos, S. I. S. Pinto

**Affiliations:** 1 CINTECX, Universidade de Vigo, Campus As Lagoas-Marcosende, 36310 Vigo, Spain; 2 Engineering Faculty of University of Porto, Institute of Science and Innovation in Mechanical and Industrial Engineering (LAETA-INEGI), Rua Dr. Roberto Frias, 4200-465 Porto, Portugal

**Keywords:** Clot, Migration risk, Cerebral aneurysm, Fluid–structure interaction, Hemodynamics

## Abstract

**Purpose:**

There are still many challenges for modelling a thrombus migration process in aneurysms. The main novelty of the present research lies in the modelling of aneurysm clot migration process in a realistic cerebral aneurysm, and the analysis of forces suffered by clots inside an aneurysm, through transient FSI simulations.

**Methods:**

The blood flow has been modelled using a Womersley velocity profile, and following the Carreau viscosity model. Hyperelastic Ogden model has been used for clot and isotropic linear elastic model for the artery walls. The FSI coupled model was implemented in ANSYS® software. The hemodynamic forces suffered by the clot have been quantified using eight different clot sizes and positions inside a real aneurysm.

**Results:**

The obtained results have shown that it is almost impossible for clots adjacent to aneurysm walls, to leave the aneurysm. Nevertheless, in clots positioned in the centre of the aneurysm, there is a real risk of clot migration. The risk of migration of a typical post-coiling intervention clot in an aneurysm, in contact with the wall and occupying a significant percentage of its volume is very low in the case studied, even in the presence of abnormally intense events, associated with sneezes or impacts.

**Conclusions:**

The proposed methodology allows evaluating the clot migration risk, vital for evaluating the progress after endovascular interventions, it is a step forward in the personalized medicine, patient follow-up, and helping the medical team deciding the optimal treatment.

**Graphical Abstract:**

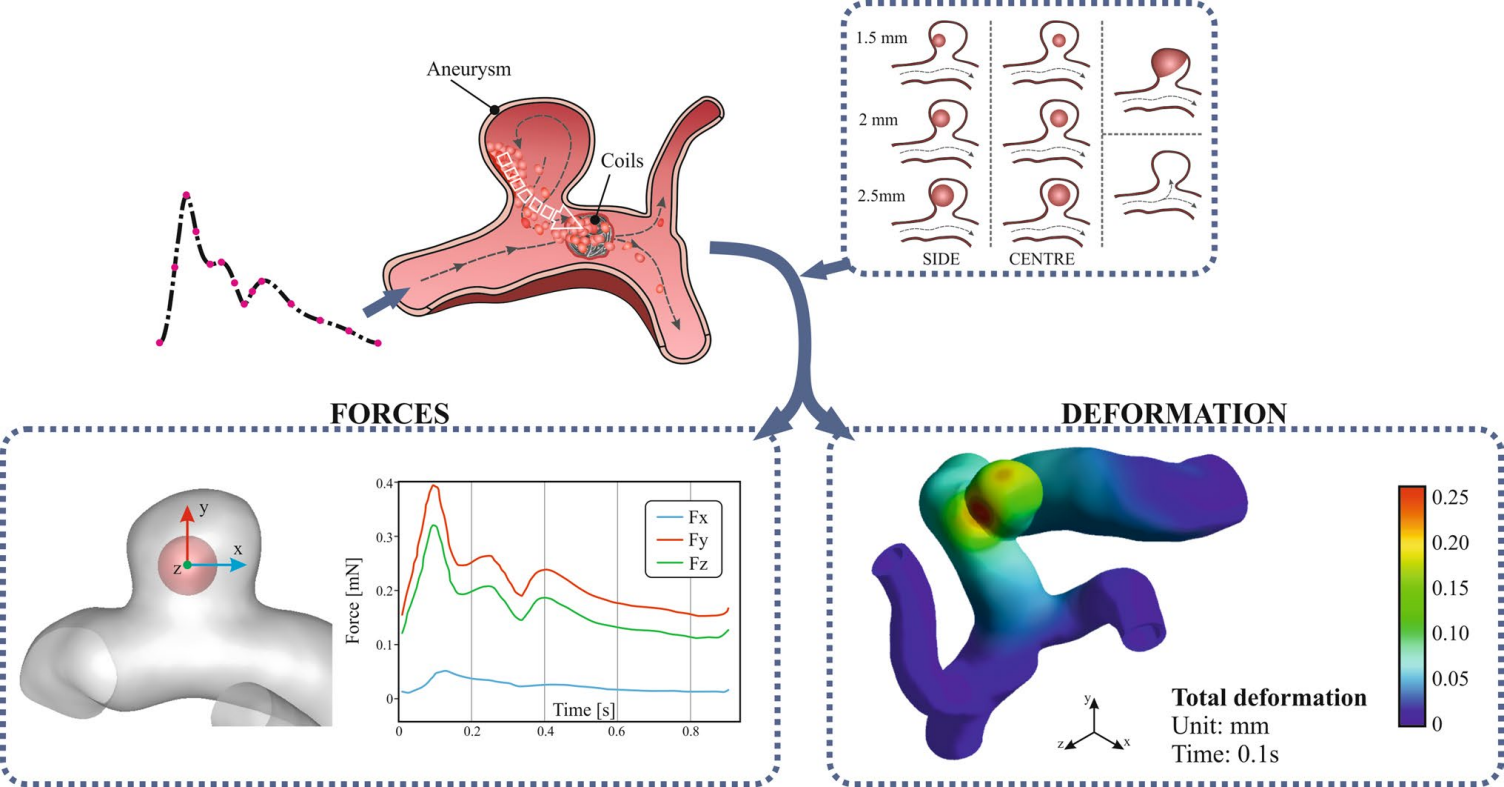

## Introduction

An aneurysm is characterized by the outward ballooning of a weakened portion of a blood vessel [1]. Aneurysms occur predominantly in arteries, at several common locations in the body. Depending on shape, size or location, there are different aneurysm classifications. Intracranial arterial aneurysm within the cerebral vasculature, is one of the most common neurovascular afflictions. The prevalence of unruptured cerebral aneurysm is between 3 and 5% of the asymptomatic general population [2], due to the small diameter of the vessels, most of them present saccular morphology [3–5]. Patients usually develop such aneurysms between 40 and 60 s, and more commonly affect females [1, 6]. Different biological, hemodynamic, predisposing genetic, environmental, and epidemiological risk factors are related with the formation of aneurysms. Due to the absence of previous symptoms, the sudden rupture of a cerebral aneurysm has a high mortality rate [7].

Endovascular coiling has become the most common treatment for cerebral aneurysms [8]. It is an effective technique for treatment of relatively wide-neck aneurysms. Delayed rupture of a previously coiled unruptured aneurysm is extremely rare [9]. In this endovascular procedure, coils are deployed in the aneurysm sac, serve as a site for platelet aggregation, enhancing the thrombosis process, and occluding the aneurysm with good clinical results. This thrombosis process is still a poorly understood phenomenon, the clot formation mechanisms are different depending on the location of the aneurysm, and there is a complex interaction between blood flow and coils. The healing process begins soon after the post-endovascular coil placement and continues for months, and involves changes at the molecular and tissue level [10].

Complications during endovascular coiling treatment still occur. Although it occurs infrequently, coil protrusion occurs occasionally [11]. There are many documented cases of migrations in different arteries, [12, 13]. The coil migration during endovascular embolization of intracranial aneurysms is a potentially serious intraprocedural complication. The majority of coil migration occurs during the procedure, 2-6% of cases [14], however there have been reports of delayed coil migration occurring after the embolization process [15], and the causes and approach are different from when migration occurs during the procedure. Although coil migration after cerebral aneurysm embolization is a rare complication, it is known that aneurysm sac growth after coil embolization, is a frequent cause of complications, and could favor the migration [16].

Thromboembolism is one of the most common complication during endovascular coiling treatment, in a range of 2-15% of incidence in these procedures. A thromboembolic event is caused by one or several clots which produces a complete or partial occlusion of arteries at the site of the aneurysm or in any other vascular territory.

Several causes have been suggested that explainable embolic sources, may be generated in the guiding catheter, on the coil wires, may be caused by the malposition of coils, or atheroma dislodged during the course of the procedure [17].

The use of in silico studies, using numerical modelling techniques (FEA, CFD, ML, FSI), is becoming more and more common in aneurysm research. Wu and Zhu [18] reviewed hemodynamic studies of intracranial aneurysms by computational fluid dynamics, CFD. Although clinical studies remain irreplaceable in aneurysm research [19, 20]. CFD has proven to be a valuable tool verifying its results with clinical data in areas such as reproducing the inflow hemodynamics of cerebral aneurysms [21], or predicting the extent of thrombus formation [22]. CFD porous media modelling has been used for simulating the hemodynamic changes in coiled aneurysms [23]. Mousavi et al. [24] used a hybrid finite-element analysis and smoothed particle hydrodynamics mix-model reconstructing a stent retriever grid, modelling the mechanical thrombectomy to the blood clot, and restoring the blood flow. Friesen et al. [25] evaluated the most common modelling approaches on aneurysms, and identified the FSI as the most realistic approach by modelling the interaction between blood flow and solid structure, thanks to the inability to reproduce changes in the vascular walls along the total cardiac cycle. The effects of endovascular coiling treatment in cerebral aneurysms have been studied by FSI, but using simplified geometrical models [26].

The modelling of cerebral aneurysms, despite the great increase in the number of studies in this field, still has an enormous development potential [27]. There are still many challenges for modelling the thrombus migration process more accurately and evaluate the migration risk in aneurysms, which can be approached in different ways. The consideration of real aneurysm morphology and artery walls, the complex structure and elastic properties of thrombus, considering coil and blood, the impact between realistic elastic solids, the coupling between fluid and the structural domains, the clot formation mechanism, or the formation of coil packing distribution, or the influence of blood viscosity using Newtonian or Non-Newtonian models on hemodynamics in coiled aneurysms are still open problems in this research field [28, 29].

**Fig. 1:**
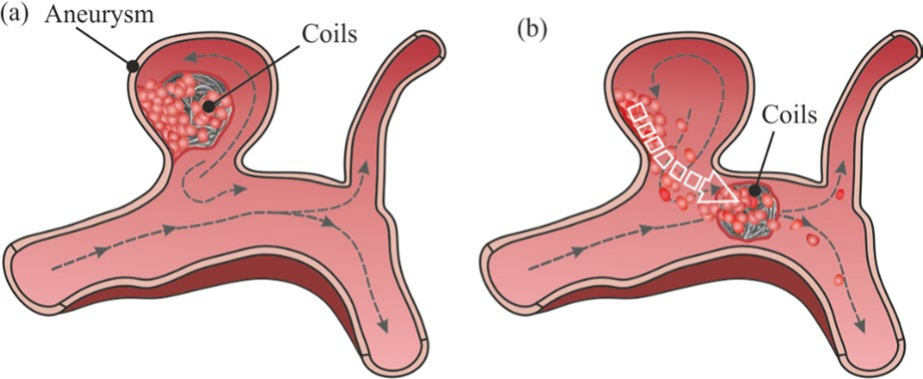
Scheme of clot migration process in an aneurysm: **a** stable coil thrombus, before migration, **b** detaching of the coil-clot, migration

To the best of our knowledge, no FSI hemodynamic analysis of forces suffered by clots inside an aneurysm has been reported before. The main novelty of the present research lies in the modelling of the aneurysm clot migration process, representative of a post-intervention situation of coiling in a realistic cerebral aneurysm, shown schematically in Fig. 1. The coil was not considered as a separate entity from the clot, and the migration process has been analysed starting from the clot detachment. A coupled FSI model, solving both the fluid and solid domains simultaneously, has been used, with pulsating transient conditions, considering the elastic properties of the clot and arterial walls, as well as the rheology of the blood. In addition, the hemodynamic forces suffered by the clot have been quantified for different sizes and positions of the clot. The coil was not fully considered in the clot-aggregate model, this research is a first step to study the clot-coil detachment.

## Methodology

### Geometric Model

**Fig. 2:**
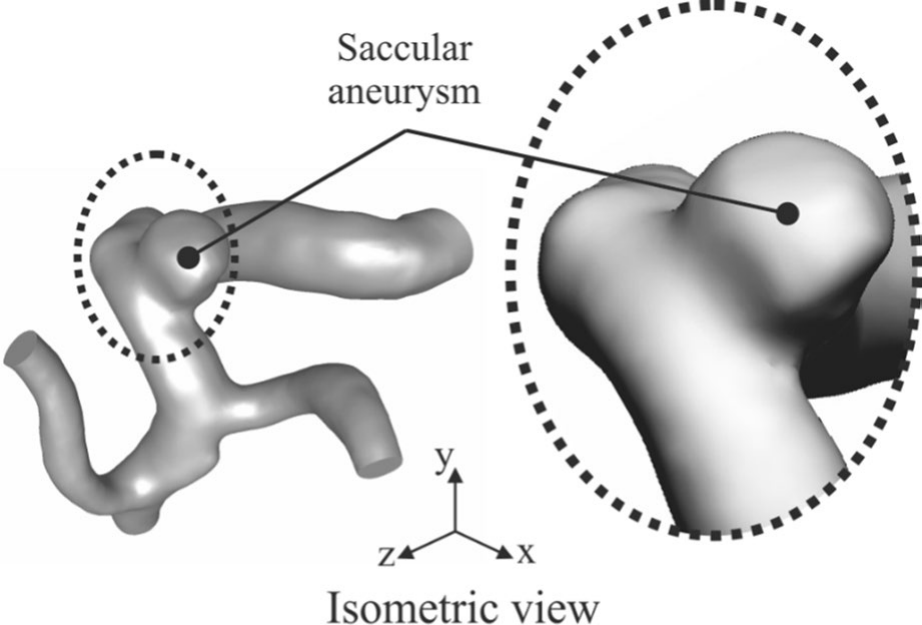
Geometry of the saccular aneurysm [30]

In this research the geometric model corresponds to a real patient aneurysm. It was selected from the open-source Aneurisk online database [30], where the model was created from DICOM images of 3D rotational angiograms. The geometry used is shown in Fig. 2. It is an Internal Carotid Artery (ICA) aneurysm, one of the most common cerebral aneurysm locations [4–6], corresponding to the internal database codification C0067.

The aneurysm is located on the side of the artery. It is a saccular type and it did not suffer rupture problems. The patient-specific case is a 42 years-old woman. The key aneurysm dimensions are a volume of 38.41mm^3^ and a surface area of 53.74mm^2^. The aneurysm neck cross section is 9.86mm^2^ and perimeter of 11.58 mm. It is a relatively wide neck, probably suitable for the endovascular management by flow diverting stents, associated with a better performance in terms of aneurysm occlusion and rate of recurrence [31]. However, studies associated this technique with an increased risk of in-stent occlusion of side branches [31], so there could be doubts about the choice of the most appropriate technique, and note that this research aims to evaluate extreme haemodynamic situations at the aneurysm site, so the traditional treatment of endosacular coiling has been considered.

The preparation of the model has been carried out using the ANSYS SpaceClaim 2020 R1 © software, a computer-aided design tool specially oriented to the repair, simplification and treatment of geometries imported in STL format. The vessel thicknesses were created from the inner wall of the vessel. In this study an artery wall thickness of 0.4 mm was used, based on the normal values ranged between 0.2 and 0.5 mm [32, 33]. The wall thickness of the aneurysm sac is 0.1 mm, considering the thinning of the thickness of the vessel’s wall, due to the dilation of the artery when an aneurysm occurs, and using a smooth transition between both regions. In order to facilitate the application of a user-defined function, UDF, created to simulate the blood inner conditions, the model has been adapted so that the inlet of the artery is circular and perpendicular to the Z axis, and coinciding with the coordinates origin.

**Fig. 3:**
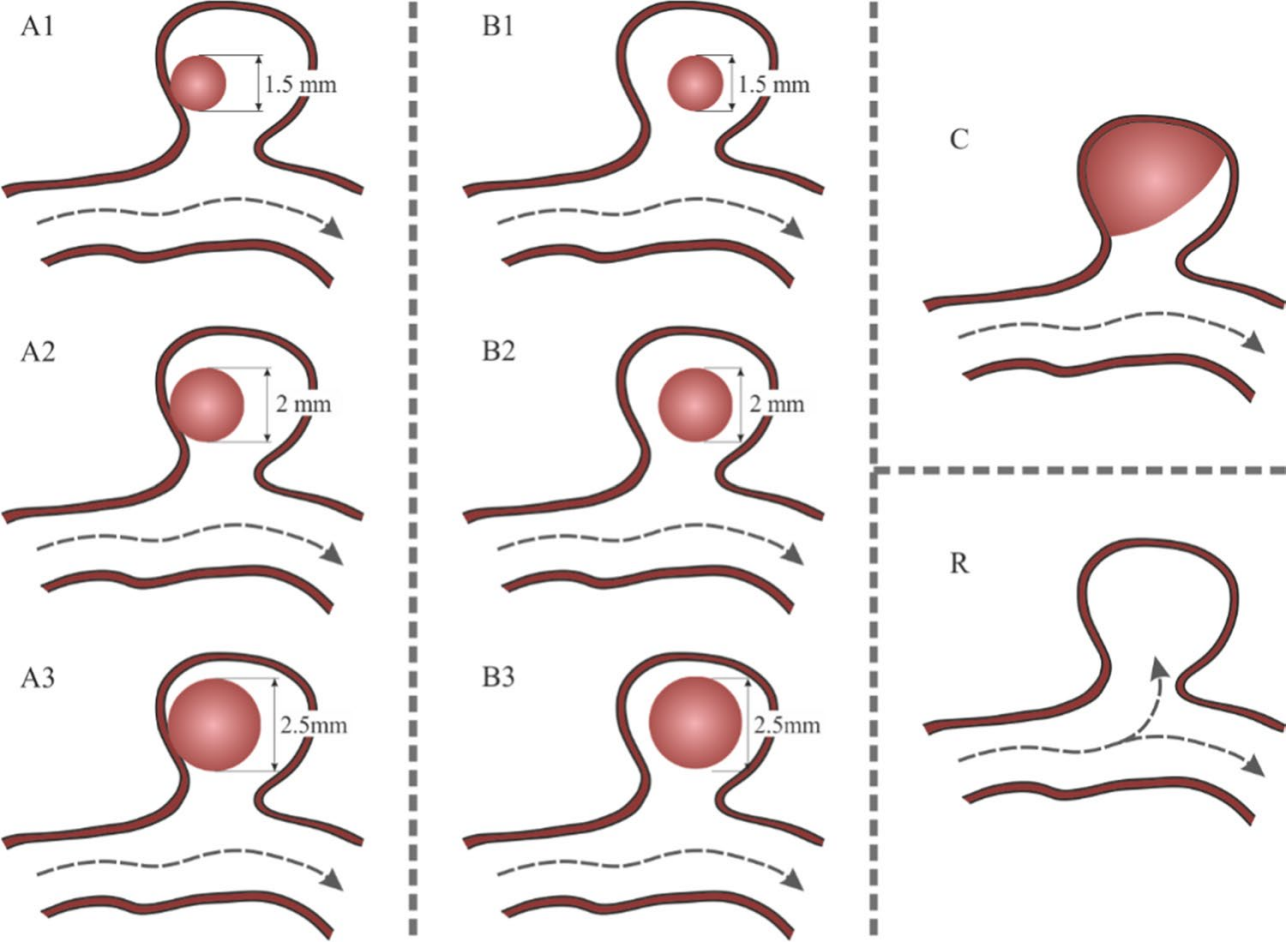
Graphic summary of clot sizes and positions modelled, including the nomenclature used

Although most of the coil procedure achieves the complete aneurysm occlusion, there is around a 11% of post-coiling embolization which only achieves a partial occlusion, [34]. Sometimes, mainly in large aneurysms, there is a residual flow within the coil increasing the risk of thrombus propagation, [17]. Therefore, in order to model the clot migrations risk, after-embolization stage, taking into account that coils have a tendency to form spherical shape packing [24, 35], and due to the uncertainty of the shape and position of the clot, a set of possible simplified cases were created, and these are detailed in Fig. 3. The first column presents three cases in which the geometry of the clot is assumed spherical, and it is in contact with the wall of the aneurysm sac, the most common position. These cases are identified with the nomenclature 'A', followed by a number: 1, 2, and 3, corresponding to the different sizes analysed: 1.5, 2, and 2.5 mm of clot diameter respectively. Similarly, it was proposed to evaluate the same three clot sizes, but located in the geometrical centre of the aneurysm, corresponding to the second column. The nomenclature used is 'B', with the same numbers criteria to specify the size of the clots. Finally, two more situations are proposed, one in which the clot occupies most of the sac volume, covering 85% of its volume, identified with the letter 'C', and the reference case, ‘R’, in which there is no clot. Following these criteria, 8 different models were built as shown Fig. 3.

### Meshing

**Fig. 4:**
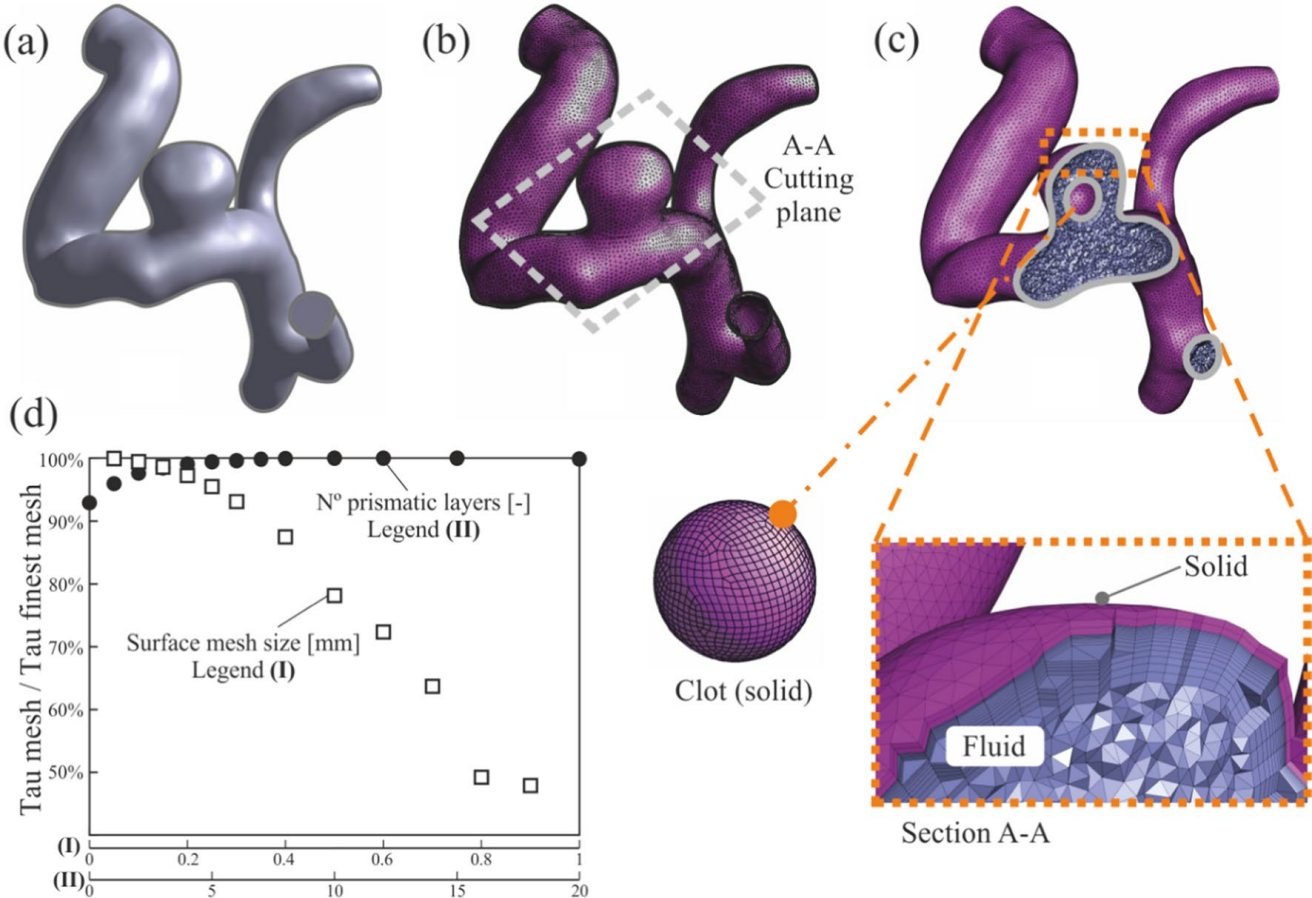
Mesh details: **a** smooth geometry, **b** surface mesh and cutting plane, **c** cut view of internal blood mesh and detail of clot mesh, **d** mesh convergence study results

The meshes employed in this research have been generated using the ANSYS® meshing pre-processing module. The mesh details are shown in Fig. 4, starting with the smooth geometry, Fig. 4a, the superficial mesh Fig. 4b, and details of the 3D mesh, Fig. 4c. The range of the meshing parameters have been selected taken into account previous studies [36, 37]. Nevertheless, a mesh convergence study was carried out in order to select the optimal meshing parameters. The simulations were performed under steady conditions, laminar model, with a constant inlet blood flow velocity 0.1 m/s, and the numerical convergence criterion was to achieve 10^-4^ in wall shear stress residual. For the mesh convergence study 12 different sizes of the surface mesh were evaluated from 0.05 to 0.9 mm, and 13 numbers of prismatic cell layers in the near-wall region, from 0 to 20. The first cell size was adjusted to obtain an average dimensionless wall distance, y + , of approximately 1, following the Ansys Fluent® recommendations [38]. In Fig. 4d, the results were evaluated using the wall shear stress ratio between results obtained for a specific mesh with the finest mesh results. Based on these results, 13 prismatic layers with a linear-growth factor of 1.15, 0.1 mm of mesh element size in clot surface, and 0.15 mm in vessels walls, were chosen as the best relationship between computational cost and precision. These meshing parameters are in the same order of magnitude as previous research [39, 40]. The average orthogonal quality was 0.85, and 0.34 the minimum value. The average skewness was 0.19, and 0.79 the maximum skewness value. These values guarantee the quality of the mesh. The solid mesh of the vessel walls was identical in all simulated cases, but the number of elements of the fluid and solid domain varies slightly due to the clot size differences. In all cases, the total number of computational cells was around 2.5 million. Finally, all these surfaces on which a boundary condition was going to be imposed were identified with a name, to facilitate the process.

## Material Properties

There are two phases in the model presented: a fluid region, corresponding to the blood, and the solid region, formed by two solids: the vessel walls and the clot, which have different properties.

Taking into account the small diameters of the arteries in this study, it is known that the non-Newtonian effects are non-negligible [41, 42], so blood has been modelled following the Carreau viscosity model, selected in several previous works [41, 43, 44]. The non-Newtonian Carreau model has been implemented through a specific User-Defined Function (UDF), Eq. (1):

1 $$\mu ={\mu }_{\infty }+\left({\mu }_{0}-{\mu }_{\infty }\right){[1+{\left(\lambda \dot{\gamma }\right)}^{2}]}^{\frac{n-1}{2}}$$

The viscosity, $\mu$, varies with the shear rate, $\dot{\gamma }$, where time constant $\lambda$ is equal to 3.313, power–law index, $n$, equal to 0.3568, zero shear viscosity, ${\mu }_{0}$, equal to 0.056 and infinite shear viscosity, ${\mu }_{\infty }$, equal to 0.00345. Blood has been assumed homogeneous and incompressible with constant density of 1050 kg/m^3^ [29, 41].

Cerebrovascular walls are formed by three concentrically arranged layers, with different thickness and properties. Loss of the vessel elasticity is a seminal event in aneurysm development, [1]. There are different options for modelling the elasticity of this multilayer wall. The cerebral vessels are less elastic than other larger arteries [45], and deformations are generally small, so some researchers have considered them as rigid walls [29, 46], and others considered them as viscoelastic material properties [47], most researchers do not consider the anisotropic behaviour of the collagen fibers and use simplified elastic or hyperelastic models [48, 49].

The model used for the artery wall material in this research is an isotropic linear elastic model governed by Hooke's Law, with 1.8 MPa for the Young’s modulus [46] and 0.45 the elastic isotropic Poisson’s ratio [26, 47]. The artery walls density was set to 1050 kg/m^3^, in the range of values used to model this tissue by other previous researches [29, 50]. Modelling the real physical properties of an intra-aneurysmal detailed coil-blood clot is very difficult due to the granular nature of the thrombus aggregate, and the random packing structure of the coil, among other uncertainties. The use of simplified properties for this thrombus is assumed as an acceptable hypothesis for this first analysis focused on the forces during the migration process. In order to assess the risk of clot migration after coiling intervention, it is essential to consider the elastic properties of the clot. It is expected that it will suffer large deformations that could be capable of changing its shape, and thus be able to cross the neck of the aneurysm. The blood clots are made up of a conglomerate of materials that give it the properties of a hyperelastic material [51]. In order to model this material, special attention was paid to the study carried out by Fereidoonnezhad et al. [52], where an extensive analysis of the different existing hyperelastic models was carried out and the results are compared with experimental data. Their results show that the Ogden model replicate the stress–strain behaviour of clots with acceptable accuracy. Considering that the aim of this research is focused on the hydrodynamic forces, not on clot deformation, the possible discrepancy associated with the volumetric behaviour is countered by the reliability and simplicity of the model. The strain energy density function for Ogden model, $\psi$, is given by Eq (2):

2 $$\uppsi =\sum_{i=1}^{N}\frac{2{\upmu }_{i}}{{{\mathrm{\alpha }}_{i}}^{2}}\left({{\overline{\uplambda }}_{1}}^{{\mathrm{\alpha }}_{i}}+{{\overline{\uplambda }}_{2}}^{{\mathrm{\alpha }}_{i}}+{{\overline{\uplambda }}_{3}}^{{\mathrm{\alpha }}_{i}}-3\right)+\sum_{i=1}^{N}\frac{1}{{D}_{i}}{\left(J-1 \right)}^{2i}$$

where μ_i_, α_i_, D_i_ are material parameters, $\overline{{\lambda }_{i}}$ are the isochoric principal stretches, $J={\lambda }_{1}{\lambda }_{2}{\lambda }_{3}$ is the Jacobian, and all the parameters are defined in Table 1.

**Table 1:** Material parameters of the Ogden model (N = 3) used.

μ_1_ (kPa)	α_1_	μ_2_ (kPa)	α_2_	μ_3_ (kPa)	α_3_	D_1_ (1/kPa)	D_2_ (1/kPa)	D_3_ (1/kPa)
0.00812	6.41	0.00248	− 5.4	0.00009	5.16	207.43	193.37	473.03

Taken from Fereidoonnezhad et al, [52]

The clot density was set to 1050 kg/m^3^, as the artery walls, thus, the mass of clots corresponding with the nomenclature 1, 2, 3, and ‘C’ are 1.86, 4.40, 8.59, and 32.1 μg respectively.

### Numerical Modelling

All arterial flows have a pulsating behaviour, associated with the cardiac rhythm, therefore, a transient simulation is required. In this research, the length needed to obtain a fully developed flow is greater than the available artery. Therefore, a fully developed flow was applied using a Womersley velocity profile [53] in the entrance of the artery, Eqs. (3) and (4) for $Q$, the input blood flow.

3 $$w\left(r,t\right)=\frac{2{B}_{0}}{\pi {R}^{2}}\left[1-{\left(\frac{r}{R}\right)}^{2}\right]+Re\left[\sum_{n=1}^{N}\frac{{B}_{n}}{\pi {R}^{2}}\left[\frac{1-\frac{{J}_{0}\left(\alpha \sqrt{n}\frac{r}{R}{i}^\frac{3}{2}\right)}{{J}_{0}\left(\alpha \sqrt{n}{i}^\frac{3}{2}\right)}}{1-\frac{2{J}_{1}\left(\alpha \sqrt{n}{i}^\frac{3}{2}\right)}{\alpha \sqrt{n}{i}^\frac{3}{2} {J}_{0}\left(\alpha \sqrt{n}{i}^\frac{3}{2}\right)}}\right] {e}^{i2\pi n\frac{t}{T}}\right]$$

4 $$Q(t)\approx \sum_{n=0}^{N}{B}_{n}{e}^{i2\pi n\frac{t}{T}}$$

where $w$ is the normal velocity, $r$ is the radius, $t$ is the instant time, ${B}_{n}$ is the complex Fourier coefficients, $R$ is the equivalent radius of the circumference with the same input area, ${J}_{0}$ is the Bessel function of the first type of zero order, ${J}_{1}$ is the Bessel function of the first type and first order, $i$ is the imaginary number, , $T$ is the cardiac cycle period, $\alpha$ is the Womersley number ($\alpha =R\surd (2\pi /vT$) which indicates that for values between 0 and 1 the flow can approach Poiseulle's law, while for values greater than 10 the flow approaches to a flat profile, and $v$ is the kinetic viscosity.

The cardiac cycle curve was obtained from the study by Ford et al. [54], where experimental measurements were made in 17 people through magnetic resonance, thus being able to obtain the points that characterize the cubic spline of the mean waveform of blood flow in a ICA.

**Fig. 5:**
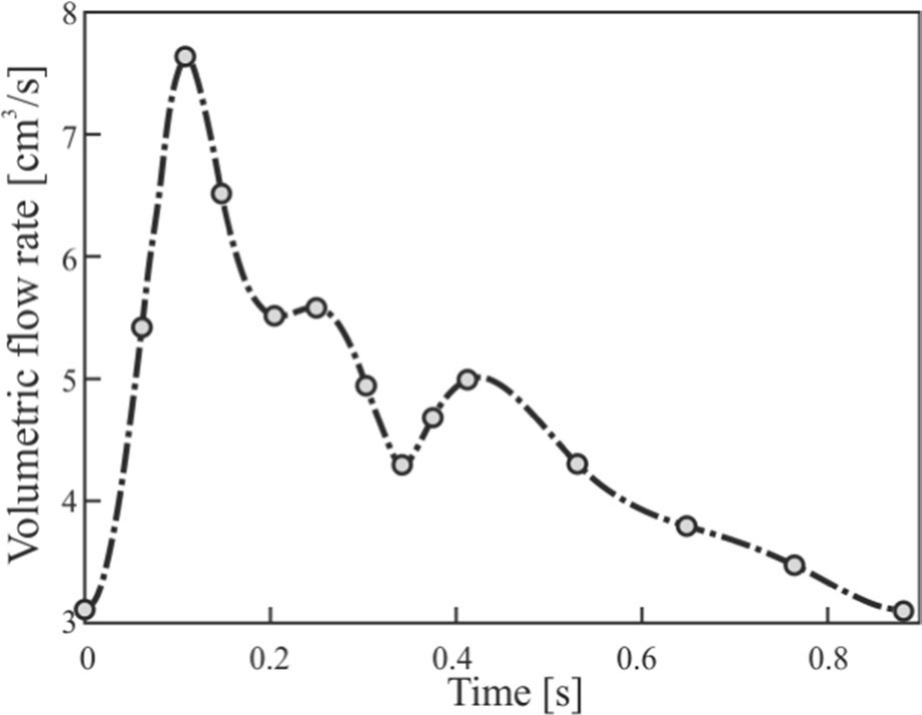
Blood flow profile corresponding to a heartbeat, applied as boundary condition in Fluent to the artery inlet

The Womersley equation has been implemented using Fourier coefficients by user-defined function (UDF), based on the UDF developed in Li's Thesis [55]. To represent the shape of the blood flow wave, the first 20 Fourier coefficients of a cubic spline have been obtained using Matlab. Figure 5 shows the characteristic points of the blood flow with the cubic spline and the flow provided by the UDF.

**Fig. 6:**
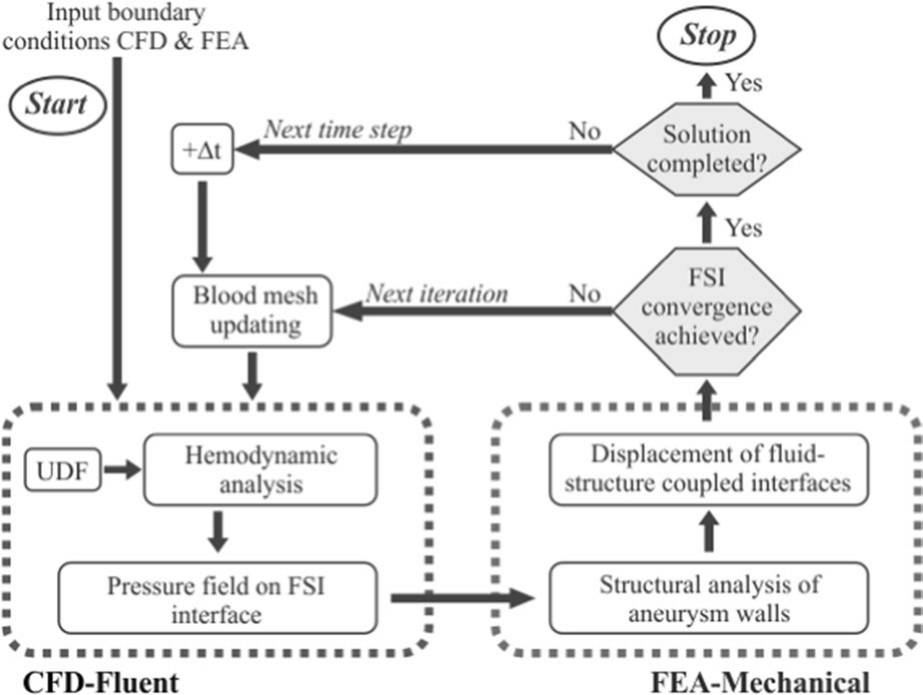
Schematic representation of the FSI simulation process [40]

The software package ANSYS® v20.2 was used for the FSI simulations. The interactions between fluid and solid domains were taken into account in the coupling mode, Fig. 6, assuming two boundary conditions: the displacement of the fluid and solid interface must be compatible, the tractions at the interface must be at equilibrium and the no-slip condition between blood and vessel has been considered [56].

In the system coupling block, responsible for the time configuration, an analysis time of 0.89 seconds with a time step of 0.001 seconds was defined, limited to 20 iterations per time step. Ten iterations have been carried out for the coupling between the two systems, for each time step. The forces calculated in the clot by Fluent are transferred by Data transfer to Transient Structural and the deformation data from Transient Structural to Fluent, using a relaxation factor of 0.99 in this coupling, to avoid the appearance of highly distorted elements. These configuration parameters have been used in previous similar studies [40], and achieve an acceptable balance between accuracy and computational cost.

The same boundary conditions were imposed for all the simulated cases. The maximum number of iterations in the structural domain for each time step of the fluid domain was 8. Concerning the fluid dynamics analysis, velocity inlet, defined in Fig. 5, was used in the artery inlet. Outlet conditions were defined with zero gauge-pressure boundary condition [57]. All the walls were set as stationary walls under the no-slip boundary condition. Point contact has been considered in simulations A1, A2 and A3, and no interaction forces between the clot and the saccular aneurysm wall have been assumed as initial conditions. Dynamic mesh was used on the walls of the artery, to the coupling system, defined as smoothing with a diffusion factor of 1.5. These parameters allow the creation of a changing mesh without loss of quality depending on the results of the deformations obtained. For the resolution of this case, the SIMPLEC algorithm has been used, with least squares cell based for gradient, second order spatial discretization setting for pressure, and second order upwind for momentum.

Each simulation was running until the scaled residual of all the variables were below 10^-4^, according to the ANSYS® Fluent convergence criterion recommendation [38]. In order to reduce the computational cost, before the coupling simulation, two previous cardiac cycles without coupling were simulated, and the results obtained were imported for the initialization of the simulation, which means a considerable reduction in the calculation time needed. The simulations were performed on an Intel® Xeon® Processor E5-2697 2.6 GHz cluster with 1280 GB of RAM, resulting in calculation times of approximately 30 hours per simulation case.

## Results and Discussion

### Hemodynamics Before Coil Procedure and Validation with Literature

In order to verify that the simulations carried out offer comparable results with previous studies, the shear stress in the walls of the artery and the aneurysm, the velocities in the neck of the aneurysm and the deformations and stresses in the artery wall and the aneurysm have been evaluated.

The time average wall shear stress (TAWSS) is defined in Eq (5), as the wall shear stress magnitude normalized by temporal-spatial along the time of the cardiac cycle. It is probably the most used parameter in aneurysm hemodynamic research.

5 $$TAWSS=\frac{1}{T}{\int }_{0}^{T}\left|\overrightarrow{WSS}\right|dt$$

**Fig. 7:**
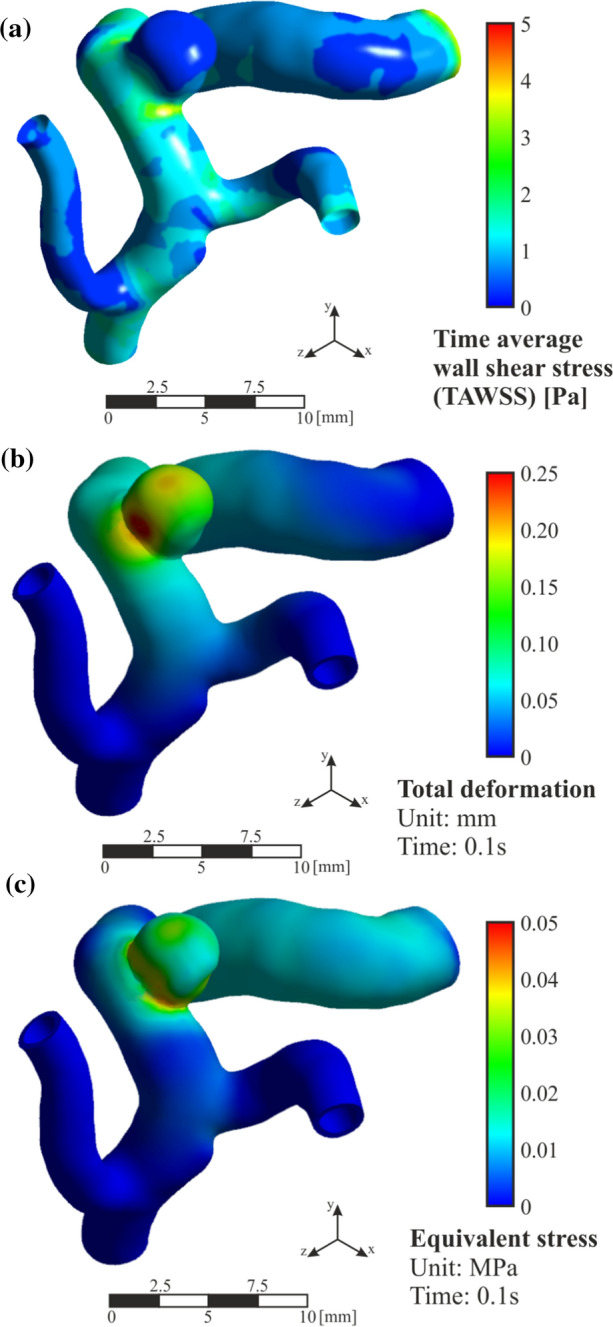
R case results. **a** Time average wall shear stress (TAWSS), **b** total deformation contours at t = 0.1 s (peak of blood flow) and **c** equivalent stress contours at t = 0.1 s

The TAWSS distribution can be seen in Fig. 7a. It is significantly lower in the aneurysm than in the rest of the artery, which can cause a risk of blood stagnation and therefore clot formation.

Analysing the values obtained, 38 Pa for the maximum wall shear stress, 0.1 Pa for the TAWSS on the sac surface, and taking into account the volume of the aneurysm, the distribution and values obtained are within the expected, results similar to previous researches [58, 59]. In Fig. 7b it is observed that the deformation produced, at the instant of peak t = 01 s, is very small, therefore forces generated in the arterial walls by the blood flow in the modelled conditions are relatively low, and the effects of artery deformation are very limited in the blood flow analysis. Contours of equivalent stress at the same time are shown in Fig. 7c, where the maximum stress is located in the region of the neck of the aneurysm, corresponding with the thinnest thickness of the arterial wall.

### Clot in Contact with Saccular Wall

**Fig. 8:**
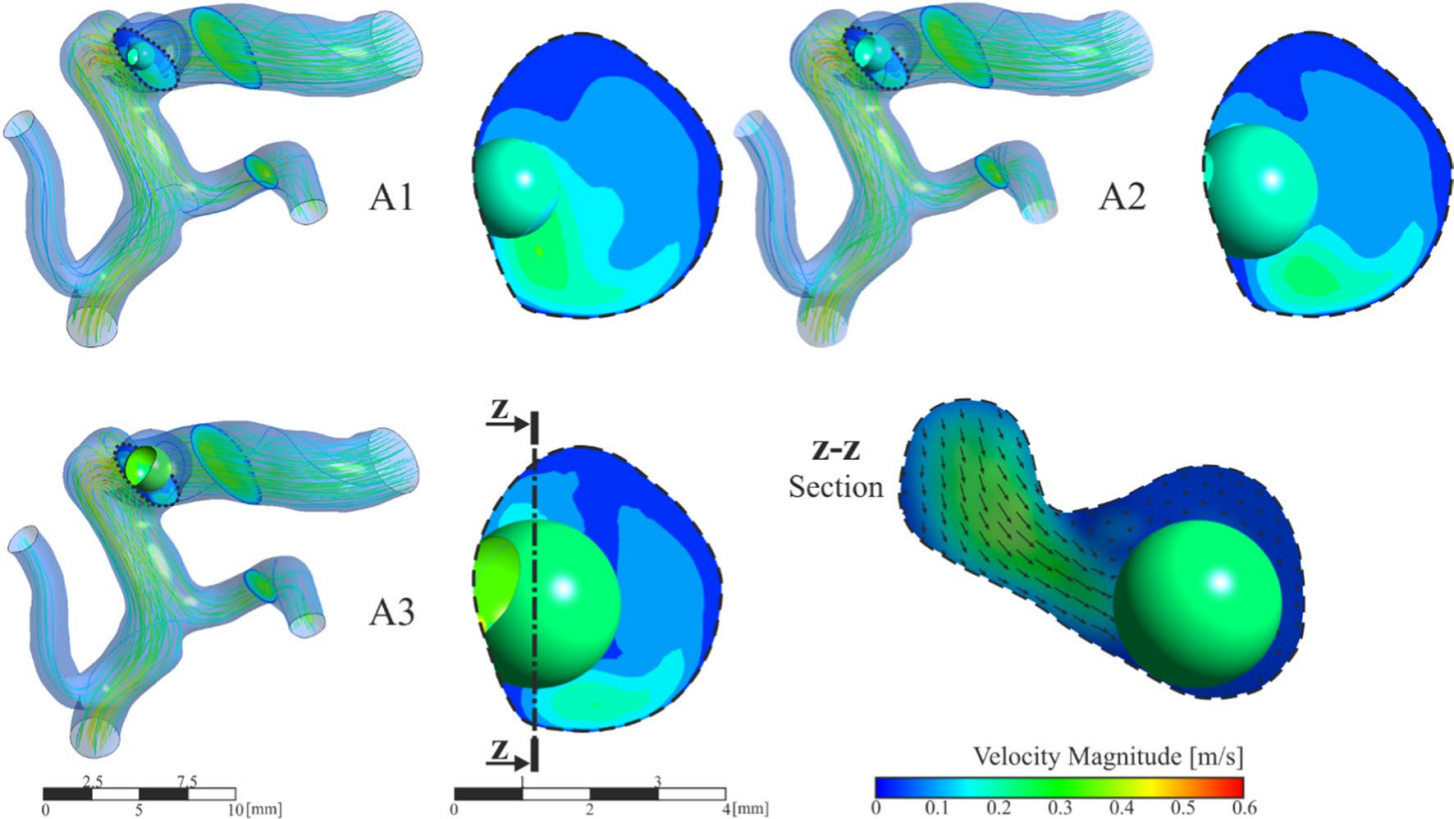
General and detailed view of velocity magnitude contours and streamlines in aneurysm region, clot in contact with saccular wall, A1, A2 and A3 simulations

This section shows the results obtained for the simulations with the clots in contact with the walls of the aneurysm, cases A1, A2 and A3. Analysing the flow within the artery, Fig. 8, the results show that the flow enters the aneurysm with the same direction as in case R. The flow impacts directly on the clot due to its position in the aneurysm. Analysing the streamlines in Fig.8, it can be concluded that part of the fluid coming from the artery collides directly with the wall of the clot, causing the resulting force direction points towards the interior of the aneurysm sac. The main stream continues its path with its subsequent collision with the wall of the aneurysm, creating a backflow that returns to the main artery causing vortices in the flow inside the aneurysm sac. These effects are identifiable in the three simulated sizes, A1, A2 and A3 by the high speed noticeable in the lower part of the detailed images on the right of Fig. 8.

**Fig. 9:**
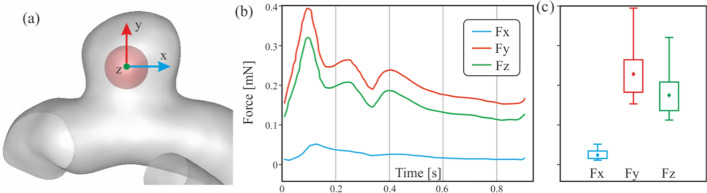
**a** Detail of the coordinate axes of the forces that affect the clot. **b** Evolution of the total force components suffered by the clot due to blood flow. **c** Corresponding statistical boxplot representation of the force components during a cardiac cycle

In order to analyse the forces on the clot due to blood flow, it is necessary to take into account the reference axes shown in Fig. 9a. Note that for the clot migration, it must be able to cross the neck of the aneurysm, the Y component of the force must be negative, so points towards the outside of the neck of the aneurysm sac, trying to detach the clot from the aneurysm towards the main artery.

Figure 9b shows the evolution of the force components during the cardiac cycle. In order to more easily compare all the results obtained, the values have been represented in graphical statistical boxplots, Fig. 9c. The statistical boxplots show: the minimum and the maximum (both whiskers); first and third quartiles (box lines), and the average (diamond).

**Fig. 10:**
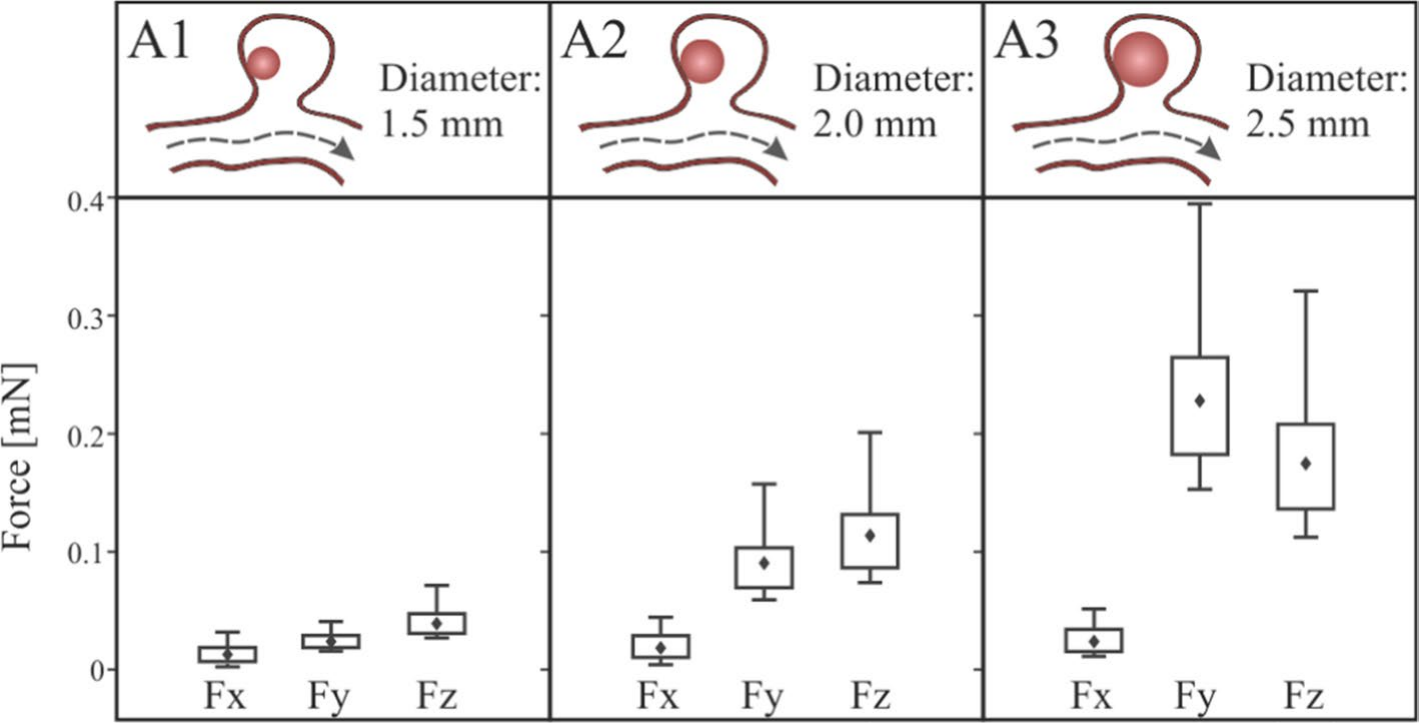
Fluid-clot force components during the cardiac cycle for clots in contact with the aneurysm wall, A1, A2 and A3 simulations

Analysing the results of the three clots A1, A2, and A3, Fig. 10, as it was expected, the x direction force component is lower than the other two in the three cases, which is consistent with the clog location in a shaded area, since X is the main direction of the blood flow in the artery. The hemodynamic forces push the clot into the aneurysm, compressing it against the walls, so it does not favour the clot migration.

A similar trend was shown for the three cases, although the bigger the clot is the stronger this force is, therefore more difficult for a clot migration to occur. An increase in clot diameter of 33% (A1→A2) implies that the hemodynamic forces are multiplied by three, with an increase of 66% (A1→A3) the hemodynamic forces are multiplied by five. In all the A-type simulations carried out, the clot never detaches from the aneurysm wall.

The force suffered by clots due to blood flow can be divided in two components, similarly to any other body surrounded by fluid: pressure force and viscosity force, as shown in Eq. (6) [60, 61].

6 $$\vec{F} = \underbrace {{\int\limits_{S} { - p\cdot\vec{n}~ds} }}_{{Pressure~force}} + \underbrace {{\int\limits_{S} {\vec{\tau }\cdot~ds} }}_{{{\text{Viscosity}}\;force}}$$

$\overrightarrow{F}$ is the total force, $p$ is the blood pressure, $\overrightarrow{\tau }$ is the wall shear stress force, $\overrightarrow{n}$ is the normal to surface vector, and $ds$ is the surface differential. The form, or pressure force, is due to pressure acting normal on the surface at all clot points, computed as the integral of the normal direction component of the pressure forces acting on all points on the clot. The skin friction force, or viscosity force is generated by the shear stresses acting on the clot surface, is due to viscosity and acts tangentially at all points on the clot surface.

**Fig. 11:**
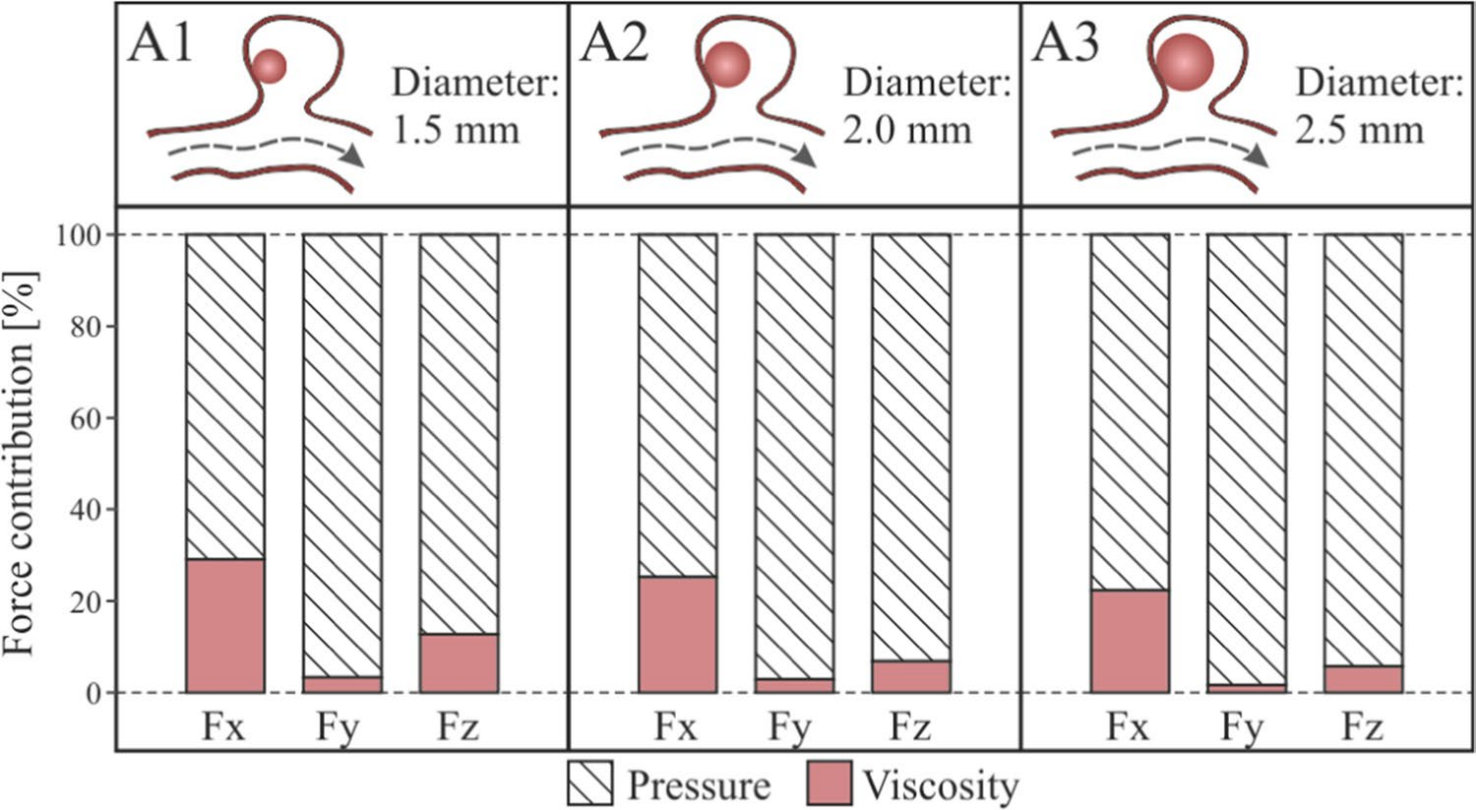
Pressure and viscosity contribution to the force for clots in contact with the aneurysm wall, A1, A2 and A3 simulations

The percentage contribution of each of these two terms to the total force has been calculated, Fig. 11. In the three cases, A1, A2 and A3, the contribution of the viscous term is lower than the contribution of the pressure term, in all the force components. Furthermore, it is shown that as the clot size increases, the contribution to the total force due to pressure increases too in the three components, x, y and z.

### Clot in the Middle of the Aneurysm

**Fig. 12:**
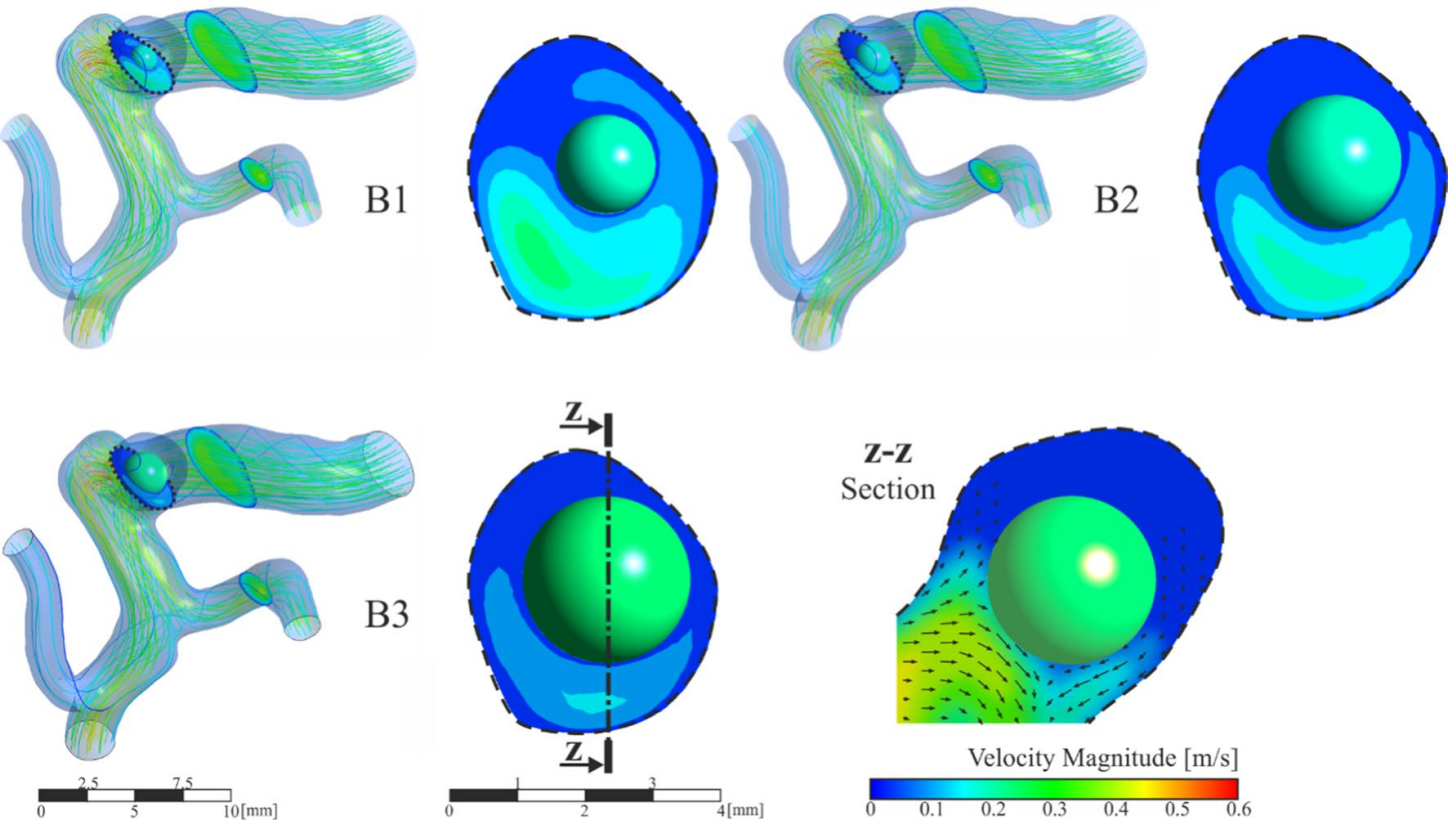
General and detailed view of velocity magnitude contours and streamlines in aneurysm region, clots in the middle of the aneurysm, B1, B2 and B3 simulations

This section analyses the situation in which the clot has been detached from the saccular wall of the aneurysm, located in the geometric centre of the aneurysm, cases B1, B2 and B3. The streamlines and velocity contours are shown in Fig. 12. Comparing the velocity fields with the previous clots in contact with the wall cases, the most notable change is that, now, there is flow surrounding the clot. Otherwise, comparing with the blood flow in the case R, where the clot was not included, a change in the velocity profiles can be noted, due to the fact that the clot blocks the aneurysm blood inflow partially. The vortices created inside the aneurysm due to the detachment of the flow from the arterial wall cause the forces incident on the clot to push it in the direction of the aneurysm neck, so increasing the risk to leaving the aneurysm sac. The blood flow in the area of the aneurysm, circulates entering through the upper part of the clot, a backwater area is produced in the deepest part of the aneurysm sac, and finally the flow leaves the aneurysm through the area located below the clot, in the three simulated sizes, B1, B2 and B3. The direction of the flow can be seen more clearly in the detail of the velocity vectors represented in the z-z section, Fig.12.

**Fig. 13:**
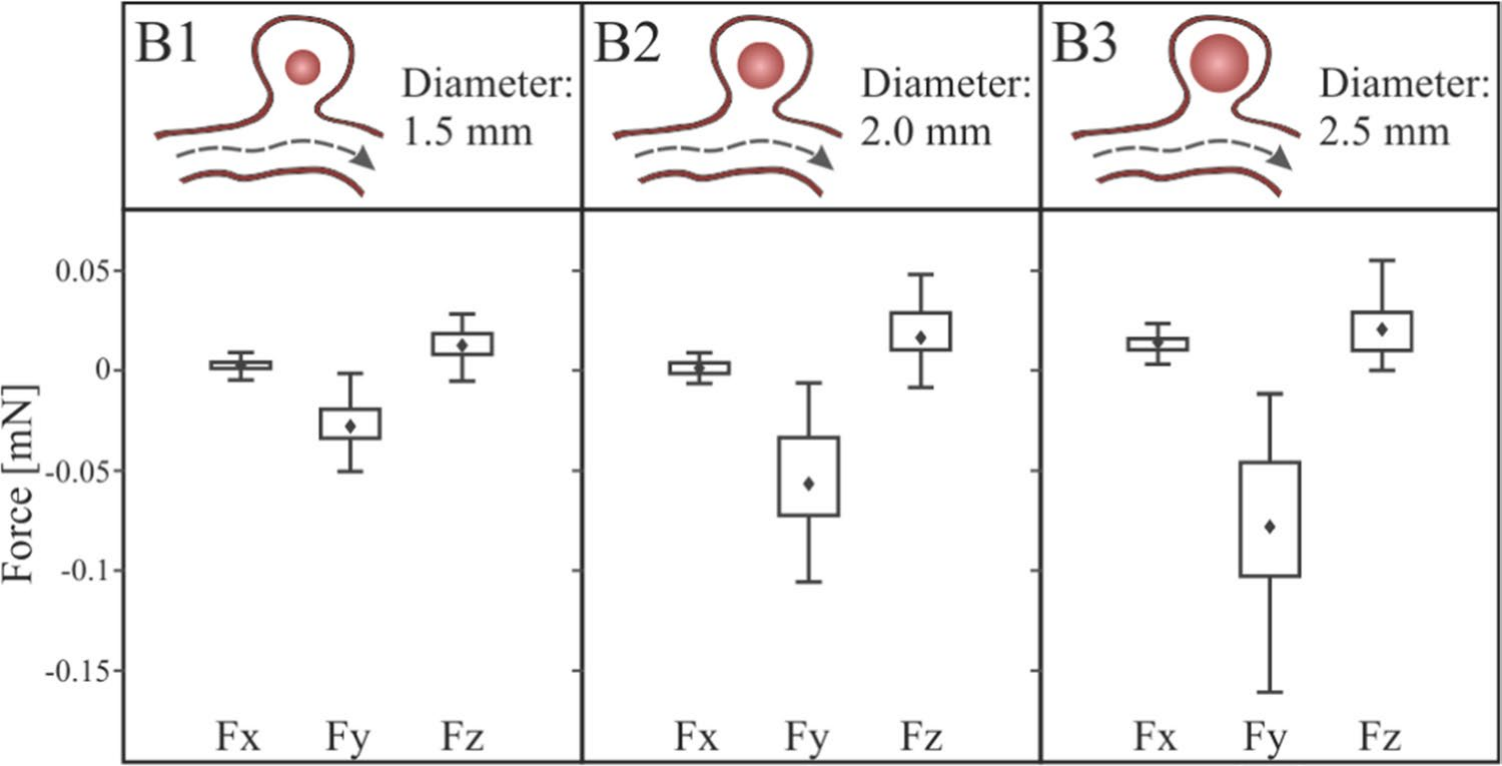
Fluid-clot force components during the cardiac cycle for clot in the middle of the aneurysm, B1, B2 and B3 simulations

In the same way as in the previous section, Fig. 9, the coordinate axes used as reference are maintained to be able to analyse the forces direction. Fig. 13 shows the force exerted by the fluid on the walls of the clot. The most important component is the force on the Y axis, if this component is negative, the force will attempt to drag out the clot from the aneurysm sac into the main artery. Analysing the results, we can conclude that, unlike what happened in the previous cases, the hemodynamic forces push the clot out of the aneurysm. In addition, the larger the clot, the stronger the forces are, increasing the risk of clot migration. An increase in clot diameter of 33% (B1B2) implies an increase in $Fy$ force of more than 100%, and an increase of 66% (B1B3) implies an increase in force of more than 180%.

**Fig. 14:**
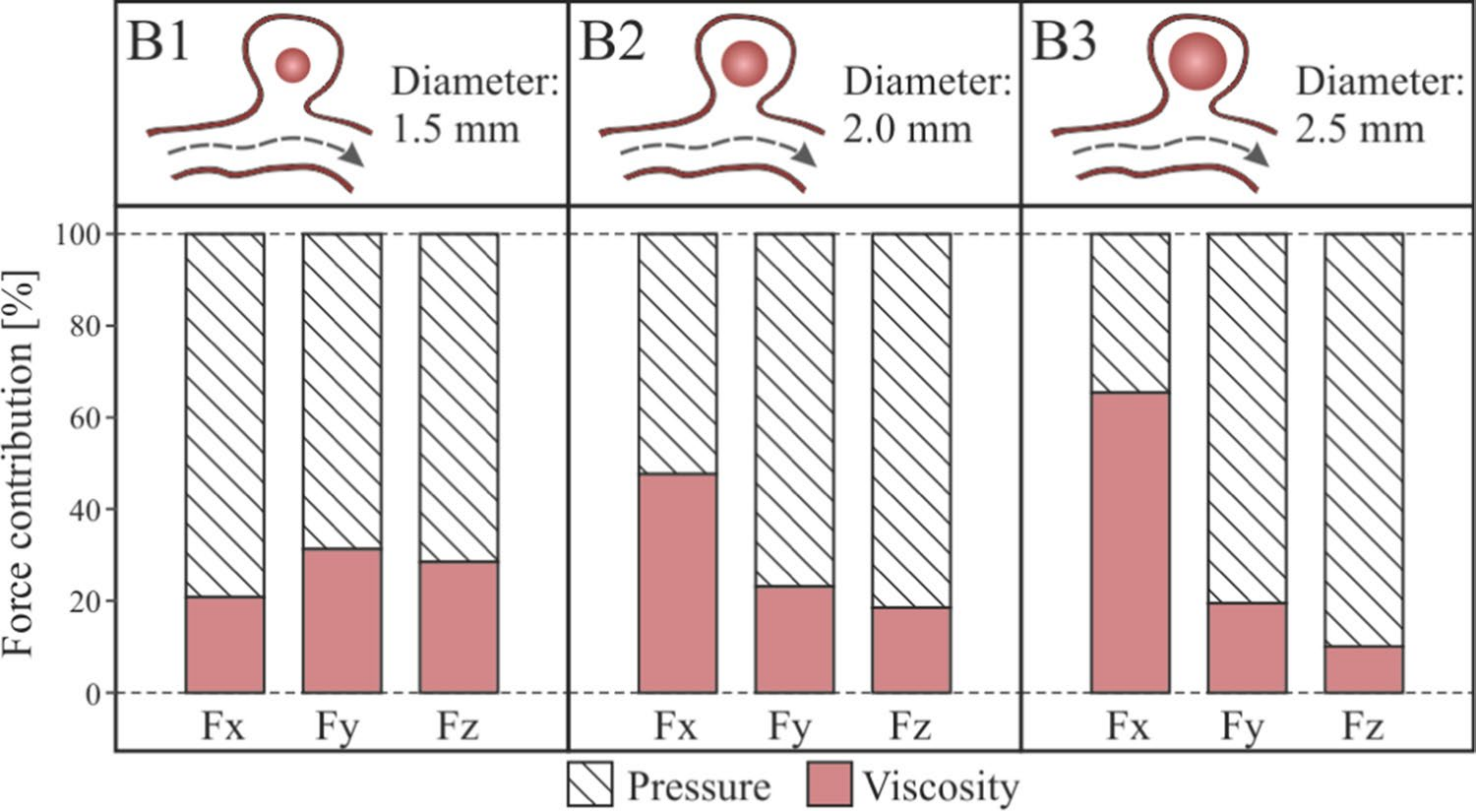
Pressure and viscosity contribution to the incident forces on the clots positioned in the middle of the aneurysm, B1, B2 and B3 simulations

The total force on the clot due to blood flow has been divided in two components, one due to pressure, and the other due to viscosity, Fig. 14. In the same way as in the case with the clot positioned in the aneurysm wall, as the size of the aneurysm increases, the x-direction contribution to the force due to pressure increases, nevertheless, the y-direction and z-direction components reduce their contribution. On the other hand, although the average contribution of viscosity to the total force is lower than the pressure contribution, it is 20% higher than in the previous A-simulations, it is due to the increase of clot surface in friction with the blood flow, and the increase of flow through the aneurysm.

Given that it is a possibility that the clot leaves the aneurysm, the duration of the simulation was extended to a sufficiently high number of cardiac cycles. Each simulation of B1, B2 and B3 configuration was repeated five times, to evaluate the behaviour of the clot, recording the clot deformation and displacement. Two cases occurred, that the clot left the aneurysm without contacting the walls (20% of the simulated cases, all of them B1-type, diameter 1.5 mm); or that the clot hit the walls of the aneurysm (80% of simulated cases). In B3 cases, diameter 2.5 mm, the clot practically blocked the neck of the aneurysm. However, the arterial walls were deformed enough to finally allow the passage of the clot. Therefore, in all B-type cases, finally the clot left the aneurysm sac after several cycles.

### Clot Blocking the Aneurysm

**Fig. 15:**
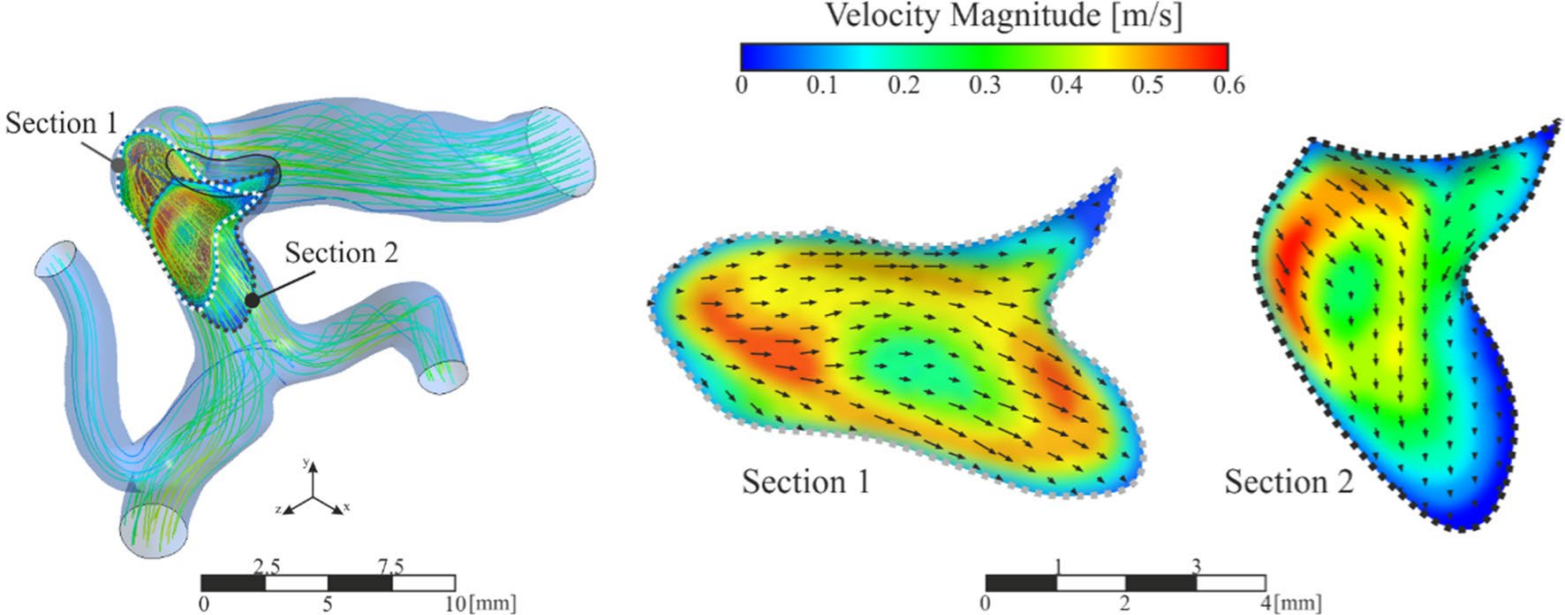
Contours of velocity magnitude and streamlines in aneurysm region, clot blocking the aneurysm, C-type

Figure 15 shows the contours and velocity vectors in the region of the aneurysm for the most realistic clot case, since it almost completely blocks the volume of the aneurysm. It can be seen that both the backwater zone and the vortices disappear, leaving only a small part of the blood flow coming from the main artery. The velocity contour indicates that there is hardly any fluid capable of pushing the clot towards the neck of the aneurysm.

**Fig. 16:**
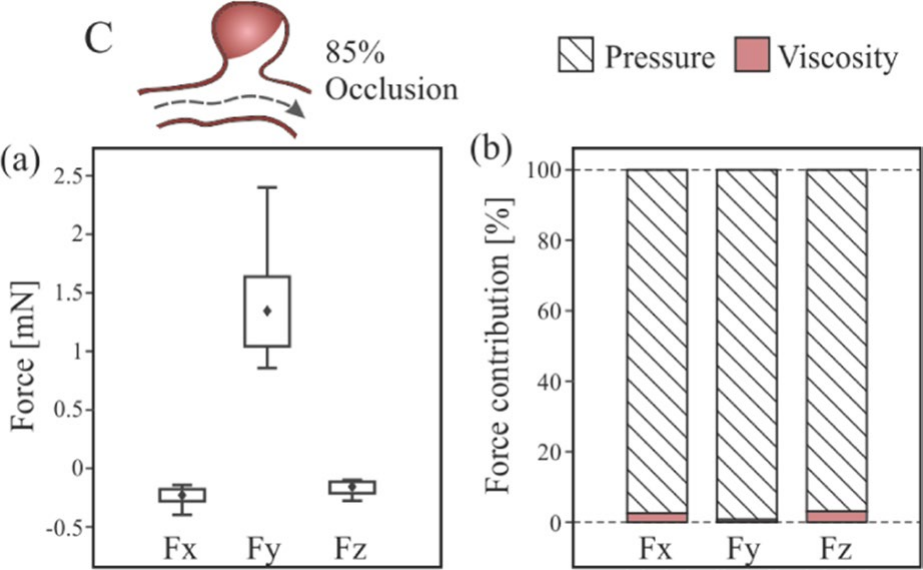
Forces for clot blocking the aneurysm, C-type, **a** fluid-clot force components during the cardiac cycle, **b** pressure and viscosity contribution to the incident force on the clot

Figure 16 shows the force components exerted by the blood flow on the clot during the cardiac cycle, and the pressure and viscosity contribution to this force. In Fig. 16a, analysing the components of this force as in previous cases, it is observed that the direction of the force on the Y axis is positive, so it points towards the neck of the aneurysm, in addition, the force magnitude is even greater than in the previous smaller spherical clots studied. Thus, it is more difficult for the clot to be able to leave the aneurysm. Fig. 16b shows the pressure and viscosity forces contribution to the incident force on the clot, and the contribution of viscosity in this case is practically negligible, lower than 3% in the three directions, because the blood flow does not surround the clot body.

There are odd situations that produce forces on the clot, that could increase the risk of migration, such as a sneeze, or a sudden impact. Given that this clot, C-type, due to its dimensions and location, is intended to reproduce the conditions closest to the clot after coiling intervention and embolization, it is of interest to evaluate the risk of detachment in these unique adverse situations.

Knowing the mass of the clot and the hemodynamic forces on the clot, calculated above, applying Newton's 2nd Law, the acceleration which would be required to detach and drag the clot from the aneurysm towards the blood flow was directly obtained. In the range of forces suffered during the cardiac simulated cycle, the range of acceleration goes from 27 to 75 m/s^2^.

The accelerations associated to a sneeze goes from 5 to 21 m/s^2^ in the most unfavorable cases [62]. Therefore, even considering the worst possible case, in which the acceleration occurs perpendicularly towards the neck of the aneurysm, the detachment conditions would not be reached, so the risk of migration remains low.

## Conclusions

In this research the clot migration process has been evaluated in a realistic geometry from an aneurysm, considering pulsatile flow, shear-thinning non-Newtonian blood flow properties, clot such as a hyperelastic material, transient simulations, by FSI coupled numerical simulations within ANSYS® software. In this research 8 different configurations were carried out, the effects of the different clot size and position inside the aneurysm were analysed separately.

Based on the results obtained, clots adjacent to aneurysm walls were pushed towards the walls due to the blood flow, making it almost impossible for them to leave the aneurysm as long as no external force is exerted on them. On the other hand, the clots positioned in the centre of the aneurysm were pushed towards the main artery due to the vortices that are generated in the volume of the aneurysm. The hemodynamic forces are very dependent on the clot size, the pushing force was multiplied by three, with an increase of clot diameter of 66%. Therefore, in these cases the migration risk exists, and depending on the clot size it might leave the aneurysm without colliding with any wall, in the smallest clot cases, or it might collide with the aneurysm walls, deform and pass though the neck and reach the main artery blood flow, completing the migration process.

The risk of migration of a typical post coiling intervention clot in contact with the aneurysm wall and occupying a significant percentage of its volume, has been evaluated. In this case, due to the blood flow forces obtained, it is concluded that the migration risk is very low. The acceleration needed to detach the clot is so high that even in the presence of abnormally intense events, associated with sneezes or impacts, the migration risk will remain very low in the case studied.

This study has provided some bases about the simulation of clot migration process, and the results obtained can lead to further studies considering other geometrical clot-aneurysm conditions. The proposed methodology is a step forward in the personalized medicine. FSI simulations of realistic clots inside aneurysms, after coiling clinical procedure, would provide an evaluating tool for patients who are at risk of suffering a clot migration event, and could help the medical team in the decision-making process.

## Abbreviations

CFD: Computational fluid dynamics
FSI: Fluid–structure interaction
FEA: Finite element analysis
ICA: Internal carotid artery
ML: Machine learning
STL: STereoLithography file
TAWSS: Time average wall shear stress [Pa]
UDF: User-defined function
WSS: Wall shear stress [Pa]
